# Tumor necrosis factor-alpha mediates the negative association between telomere length and kidney dysfunction

**DOI:** 10.7150/ijms.87254

**Published:** 2023-09-25

**Authors:** Mengya Qi, Jie Yu, Fan Ping, Lingling Xu, Wei Li, Huabing Zhang, Yuxiu Li

**Affiliations:** Department of Endocrinology, Key Laboratory of Endocrinology, Ministry of Health, Peking Union Medical College Hospital, Peking Union Medical College, Chinese Academy of Medical Sciences, Beijing 100730, China.

**Keywords:** Leukocyte Telomere Length, Renal Dysfunction, Albuminuria, Renal Function Decline, Hypertension, TNF-α

## Abstract

**Aim/hypothesis:** The relationship between peripheral blood leukocyte telomere length (LTL) and kidney dysfunction, especially in people with hypertension, remains unclear. No clinical study has explored the role of inflammation and oxidative stress in the relationship between LTL and kidney dysfunction. Therefore, we examined the relationship between baseline LTL and albuminuria progression and/or rapid renal function decline in Chinese patients with or without hypertension and investigated whether inflammation and oxidative stress played a mediating role in this relationship.

**Methods:** We conducted a prospective study including 262 patients in a 7-year follow-up period from 2014 to 2021. Data on LTL, inflammation, oxidative markers, renal function, and urine protein levels were assessed. Kidney dysfunction was defined as either albuminuria progression, rapid decline in renal function, or the composite endpoint (albuminuria progression and rapid decline in renal function). Logistic regression and simple mediation models were used for the analysis.

**Results:** In this cohort (mean age, 54.3±9.7 years; follow-up period, 5.9±1.1 years), 42(16.0%), 21(8.0%), and 59(22.5%) patients developed albuminuria progression, rapid eGFR decline, and the composite endpoint of kidney dysfunction, respectively. Logistic regression analysis showed that each standard deviation decrease of baseline LTL and the lower quartile (Q) of baseline LTL were significantly correlated with an increased risk of rapid decline in renal function (OR=1.83 [95% CI 1.07, 3.27] per 1SD, *P*=0.03; OR=7.57 [95% CI 1.25, 145.88] for Q1 vs. Q4, *P* for trend=0.031); and the composite endpoint of kidney dysfunction (OR=1.37 [95% CI 0.97, 1.96] per 1SD, borderline positive *P*=0.072; OR=2.96[95% CI 1.15, 8.2] for Q1 vs. Q4, *P* for trend=0.036). The mediating analysis showed that tumor necrosis factor (TNF)-a partly mediated the relationship between LTL and rapid decline in renal function (direct effect: β=0.046 95%CI [0.006, 0.090],P=0.02; indirect effect: β=0.013 95%CI [0.003, 0.020]), and the mediating proportion was 22.4%.In subgroup analyses, LTL was inversely associated with rapid decline in renal function or the composite endpoint of kidney dysfunction only in patients with hypertension (OR=49.07[3.72,211.12] *vs.*1.32[0.69,2.58] per 1SD, *P* for interaction=0.045;OR=3.10 [1.48, 7.52] *vs.*1.08[0.92,1.63] per 1SD, *P* for interaction=0.036).

**Conclusion:** Baseline LTL could independently predict kidney dysfunction at follow-up, especially in participants with hypertension. TNF-a partially mediated the negative association between LTL and kidney dysfunction.

## Introduction

Telomeres are tandem repeats of the eukaryotic chromosomal terminal DNA sequence TTAGGG. Leukocyte telomere length (LTL) progressively shortens with each cell division, and telomeres attrition is a hallmark of cellular aging. Additionally, LTL has been associated with cardiovascular diseases, diabetes, cancer, and chronic kidney disease (CKD) 1.

LTL is associated with CKD and end-stage renal disease (ESKD) development, and albuminuria progression 2-6. A Russian study showed a significant relationship between albuminuria levels and telomere length 7. However, the association between LTL and the declining estimated glomerular filtration rate (eGFR) remains inconclusive. In recent years, genomic and epigenetic studies have found that telomere-related genes and pathways may be a novel biomarkers or therapeutic targets for kidney disease. to find can treat DKD 8. Claire Hill et al. also found that differential methylation of telomere-related genes is associated with kidney disease in individuals with type 1 diabetes 9. Olutope Arinola Akinnibosun et al. put forward that preserving telomere length as a potential therapy for CKD 10.

However, our previous study did not find the relationship between LTL and eGFR 11. A study in the United States showed that decreased kidney function was associated with shorter telomere length at baseline and faster telomere shortening. However, these associations are entirely explained by advancing age 12. The rate of eGFR decline is a useful indicator of kidney dysfunction; and an annual eGFR decline of 3.3% or more, defined as 'rapid decline,' has been associated with a higher risk of developing ESKD 13. Prospective studies have reported a high incidence of a rapid decline in eGFR in patients with diabetes with short telomeres 14. However, in another prospective study during 8 years of follow-up, buccal telomere length was not associated with the changing rate of eGFR 15.

The most common causes of CKD are poorly controlled diabetes and hypertension 16. However, the relationship between LTL and kidney dysfunction has mostly been studied in general populations and participants with either diabetes, heart failure, or cardiovascular risk 3, 17-19. Hypertension causes small renal artery sclerosis, interstitial inflammatory fibrosis, and tubular atrophy or loss, thereby resulting in renal damage or decreased renal function 20. A meta-analysis of 16 studies showed that patients with hypertension and prehypertension have an increased risk of decreased eGFR 21. However, the relationship between LTL and kidney dysfunction, a leading cause of CKD, has not been studied in participants with hypertension.

Moreover, the mechanisms underlying the association between LTL and kidney dysfunction remain unclear. Some animal studies have shown that chronic inflammation and oxidative stress play important roles in LTL shortening as well as kidney dysfunction 22-24. Telomere dysfunction is associated with impaired mitochondrial biogenesis and function, increased reactive oxygen species and inflammatory immune response, leading to kidney dysfunction 25, 26. However, previous clinical studies that reported the relationship between LTL and kidney dysfunction failed to explore the role of inflammation and oxidative stress because of the lack of data on these indices 5, 19. Therefore, it is necessary to examine these markers to study the role of inflammation and oxidative stress between LTL and kidney dysfunction.

The purpose of our study was to examine the relationship between baseline LTL and albuminuria progression and/or rapid renal function decline during follow-up in patients with or without hypertension in rural Chinese communities and to investigate whether inflammation plays a mediating role in this relationship.

## Methods

### Study population

The current prospective study, which included a Chinese cohort from the suburb of Changping, Beijing, was conducted between March 2014 and July 2021, during a mean follow-up of 5.97±1.16 years. The research protocol and consent procedures were approved by the Ethics Committee of the Peking Union Medical College Hospital. All the participants provided written informed consent. After excluding participants who lacked baseline LTL results, albuminuria results, serum creatinine results, and had a baseline urine albumin-creatinine ratio [ACR] ≥300 mg/g Cr or eGFR <60 mL/min/1.73 m^2^, 262 participants were included in this study.

Demographic data collected at baseline included age; sex; history of use of anti-glycemic, antihypertensive, and anti-lipid drugs; and history of diabetes, hypertension, and hyperlipidemia. Anthropometric data, including height, weight, waist circumference (WC), hip circumference, systolic blood pressure (SBP), and diastolic blood pressure (DBP), were collected. The formula for calculating the body mass index (BMI) was height (in meter)/weight (in kilogram) squared. Overweight and obesity were defined as 24 kg/m^2^ ≤BMI <28 kg/m^2^ and BMI ≥ 28 kg/m^2^, respectively.

Urine and blood samples were collected from participants during follow-up to assess albuminuria status and renal function progression. According to the World Health Organization criteria, normal glucose tolerance (NGT) was identified using the 75-g oral glucose tolerance test: fasting plasma glucose (FPG) <6.1 mmol/L and 2-hour post-load plasma glucose (2h-PG) <7.8 mmol/L. Pre-diabetes was indicated by impaired fasting glucose measured as 6.1 mmol/L ≤ FPG <7.0 mmol/L and 2h-PG <7.8 mmol/L, impaired glucose tolerance (IGT) measured as 7.8 ≤2h-PG <11.1 mmol/L and FPG <6.1 mmol/L, or both impaired fasting glucose and IGT. Diabetes was indicated by FPG ≥7.0 mmol/L or 2h-PG ≥11.1 mmol/L.

### Calculations of eGFR and ACR

eGFR was calculated using the Chronic Kidney Disease Epidemiology Collaboration equation, based on the measurement of serum creatinine and anthropometric data, such as age and sex. ACR was calculated based on urine microalbumin and urine creatinine levels. The equations are as follows:

eGFR=142 × min (standardized S_cr_/K,1)^ α^ × max (standardized S_cr_/K,1)^-1.200^ × 0.9938^Age^ × 1.012 [if female] mL/min/1.73 m^2^

where K=0.7 (female) or 0.9 (male), α= -0.241 (female) or -0.302 (male), and ACR=urinary albumin/urinary creatinine (mg/g Cr).

### Measurement of LTL

LTL assays were performed using the blood samples collected at baseline. The details of the LTL measurements are described in our previous publication 27. LTL was determined, as the ratio of the telomere repeat copy number to the single copy number (T/S ratio), using a monochrome multiplex quantitative polymerase chain reaction protocol. z scores of log transformed LTL were calculated.

### Assessment of oxidative stress and inflammatory markers

Oxidative stress and inflammatory marker levels were measured using the blood samples collected at baseline. TNF-α, interleukine-6(IL-6), 8-hydroxy-2 deoxyguanosine(8-OHDG); superoxide dismutase (SOD); glutathione reductase (GR) concentrations were determined using an ELISA kit (Cloud-Clone Corp, Houston, USA) by the Beijing Institute of Biotechnology.

### Definition of hypertension

Hypertension was defined either as having been diagnosed with hypertension, taking antihypertensive drugs, or SBP ≥140 mmHg and DBP ≥90 mmHg at rest.

### Definition of the outcomes

Kidney dysfunction was defined according to the following: (1) A rapid decline in eGFR: A rapid decline in eGFR was defined as decline in eGFR of ≥3.3% per year 28, 29. Percentage change per year of eGFR was calculated as follows: a total of 262 participants with at least two eGFR measurements during follow-up were included in the calculation of the eGFR slope. Linear mixed-effects regression was used to calculate the eGFR slope for each individual, which was then re-expressed as the percentage change per year of eGFR. (2) Albuminuria progression was defined as the development of either microalbuminuria or macroalbuminuria from normal albuminuria at baseline, or macroalbuminuria from microalbuminuria at baseline. (3) The composite endpoint of kidney dysfunction:a rapid decline in eGFR and/or albuminuria progression.

### Statistical analysis

Participants were divided into quartiles (Q) based on their baseline LTL levels. Continuous variables with normal distribution are expressed as mean ± standard deviations (SDs), whereas non-normally distributed variables are presented as median and interquartile range. Categorical variables are expressed as numbers with corresponding percentages. Comparisons between groups were performed using a one-way analysis of variance for continuous variables and the χ^2^ test for categorical variables. Bonferroni correction was used for post-hoc comparisons.

The associations between LTL and the outcomes were analyzed using logistic regression analysis, with odds ratios (OR) and associated 95% confidence intervals (CIs) computed for quartiles of baseline LTL and one SD change in baseline LTL. For the computation of ORs for one SD decrease in baseline LTL, a minus z-score for log transformed LTL was calculated for each participant. Four models were generated: Model 1: not adjusted. Model 2: adjusted for age, sex. Model 3: adjusted for Model 2 +BMI, HbA1c, SBP, DBP, LDL-C, TG, UA. Model 4: adjusted for Model 3+ baseline ACR,baseline eGFR.

To explore whether oxidative stress and inflammatory markers mediated the effect of LTL on kidney dysfunction, R package(mediation) was used for mediation analysis, and all mediation models were adjusted for age, sex, BMI, HbA1c, SBP, DBP, LDL-C, TG, UA, baseline ACR and baseline eGFR.

A two-sided P<0.05 was considered statistically significant. Statistical analysis was performed using IBM SPSS Statistics for Windows, version 26.0 (IBM Corporation, Armonk, NY, USA) and R for Windows, version 4.2.3.

## Results

### Baseline characteristics of the study populations

The baseline characteristics of participants are shown in Table 1. Participants in the lower LTL quartiles had a lower eGFR, higher TNF-a and higher SOD than those in the other quartiles (*P<*0.05). However, age, sex, BMI, WHR, SBP, DBP, FPG, HbA1_C_ TC, TG, HDL-C, LDL-C, IL-6, 8-OHdG, and GR did not differ between the four groups (*P*>0.05). Additionally, the percentages of participants with microalbuminuria, diabetes, hypertension, hyperlipidemia, hyperuricemia, and obesity were not significant different between groups (all* P*>0.05).

### Associations of baseline LTL with the risk of albuminuria progression and/or rapid decline in renal function

During the follow-up period, 42 (16.0%) participants developed albuminuria progression,21 (8.0%) participants had a rapid decline in renal function,and 59 (22.5%) participants reached the composite endpoint of kidney dysfunction (albuminuria progression and /or rapid decline in renal function). Associations of baseline LTL with the risk of albuminuria progression and/or rapid decline in renal function are shown in Table 2. After adjusting for traditional risk factors, LTL was significantly inversely correlated with rapid decline in renal function and the composite endpoint of kidney dysfunction, but not with albuminuria progression.

With each 1 SD reduction in LTL, the risk of rapid decline in renal function increased by 83% (OR=1.83 [95% CI 1.07, 3.27]; *P*=0.03) (Table 2). When LTL was modeled as quartiles, the ORs of rapid decline in renal function increased with LTL quartiles decreasing; and the OR was 7.57 for Q1 versus Q4 of LTL in the fully adjusted Model 4 ([95% CI 1.25, 145.88], *P* for trend=0.031) (Table 2).

With each 1 SD decline in LTL, the risk of composite endpoint of kidney dysfunction increased by 37% (OR=1.37 [95% CI 0.97, 1.96]; borderline positive *P*=0.072), and the OR was 2.96 for Q1 versus Q4 of LTL ([95% CI 1.15, 8.2], *P* for trend=0.036) (Tables 2).

### Mediation models of the association among inflammation and oxidative stress indicators, LTL, and kidney outcome

We further tested whether inflammation and oxidative stress indicators mediated the association between LTL and rapid decline in renal function or the composite endpoint of kidney dysfunction.

In the mediation analysis, LTL was found to have both significant direct and indirect effects on rapid decline in renal function (direct effect: β=0.046 95%CI [0.006, 0.090)]; indirect effect: β=0.013 95%CI [0.003, 0.020]), whereas TNF-α partly mediated this negative effect of LTL on rapid decline in renal function and the proportion was 22.4% (Table 3). Mediation analysis between LTL and the composite endpoint of kidney dysfunction did not reach a significant level (Table 3). Moreover, we also tested other inflammation and oxidative stress indicators including IL-6, 8-OHdG, SOD and GR, and did not find these indicators played a mediating role in the associations (Supplementary Table 1).

### Associations of baseline LTL with the risk of albuminuria progression and/ or rapid decline in renal function in participants with or without hypertension

Among 262 participants, 81 had hypertension. Subgroup analysis showed that the negative associations between LTL and rapid decline in renal function or the composite endpoint of kidney dysfunction were only significant in participants with hypertension after adjusting for confounding factors (OR=49.07 [95%CI 3.72, 211.12], *P* for interaction=0.045; OR=3.10 [95%CI 1.48, 7.52], *P* for interaction=0.036) (Table 4). The associations between LTL and albuminuria progression was not significant neither in participants with hypertension or without hypertension.

In addition, we also conducted mediation analysis among TNF-a, LTL and rapid decline in renal function or the composite endpoint of kidney dysfunction in participants with hypertension, and no mediating effects were found by TNF-a (Table 3).

## Discussion

In this prospective study of 262 adults in a rural Chinese community, we demonstrated an independent negative association between baseline LTL and kidney dysfunction (including rapid decline in renal function and the composite endpoint of kidney dysfunction) during follow-up. Additionally, we showed that TNF-α partly mediated the association between LTL and rapid renal function decline. Lastly, we found a negative association between baseline LTL and kidney dysfunction (rapid decline in renal function and the composite endpoint of kidney dysfunction), which remained significant only in participants with hypertension in the subgroup analysis.

This study posed three central questions: 1) whether there was an association between LTL and kidney dysfunction; 2) whether oxidative stress and inflammation played a mediating role in the association between LTL and kidney dysfunction; and 3) whether the association between baseline LTL and kidney dysfunction was affected by hypertension status.

For the first question, we found a negative association between LTL and the composite endpoint of kidney dysfunction, which is consistent with previous studies. A prospective study including 132 individuals with type 1 diabetes and another study including 691 individuals with type 2 diabetes showed that telomere length is associated with albuminuria progression 6, 30. In addition, an American study with 889 participants (a third of whom had diabetes) and another study with 4085 participants with type 2 diabetes showed that LTL was negatively associated with CKD progression (defined as advanced renal failure or requiring renal replacement therapy [dialysis treatment or kidney transplantation]) 3, 14. Furthermore, we also found a negative relationship between LTL and rapid decline in renal function, a finding that was not always consistent with that of previous studies. The Mild to Moderate Kidney Disease (MMKD) study including 166 patients with CKD at a median follow-up of 4.5 years found that LTL was shorter in patients who exhibited doubling of baseline serum creatinine levels 30. Another study including 3964 participants at a follow-up of 8 years found that participants with shorter buccal telomere lengths were more likely to have a normal-to-impaired kidney function trajectory 15. Recently, a large and long-term follow-up study of 4085 Chinese individuals with type 2 diabetes showed robust results, indicating that shorter LTL at baseline is associated with a rapid decline in eGFR (>4% per year) during 14 years follow-up 3, which directly supports our results. Similarly, However, a study including 151 patients with type 1 diabetes and a follow-up of 11.1 years did not find any association between telomere length and the rate of decline in eGFR 15. Similarly, another study found that telomere length was not associated with the rate of change in eGFR over a follow-up of 8 years in healthy individuals 31. However, other studies have found that telomere length is associated with decreased eGFR and lower mortality in patients with CKD, and telomere therapy may be useful for the treatment of CKD 10, 32.

Regarding the second study question, our result suggests that LTL shortening increases the risk of rapid decline in renal function, in which TNF-a plays a mediating role. The relationship between telomeres and inflammation is complex. Inflammation can lead to telomeres dysfunction and cause telomeres shortening 33, 34, whereas telomeres shortening can cause an inflammatory response 35. In *in vitro* experiments, the cells with the shortest telomere exhibited the highest levels of TNF-a expression 36. Animal studies have also shown that TNF-a levels are increased in animals with shorter telomeres 37. An *in vitro* experiment revealed that short telomeres can mediate additional downstream activation of ZBP1(S), via TERRA, to form ZBP1 filaments on the mitochondria, thereby promoting the inflammatory cycle 38. In contrast, other animal experiments have shown that inflammation and oxidative stress significantly contribute to renal damage associated with hypertension, and cytokines, such as interferon-γ, TNF-a, and IL-17, affect Na+/H+ exchangers in the kidney 22. In addition, blocking TNF-a using a murine anti-TNF-a antibody confers kidney protection compared to that in a vehicle-treated Ins2^Akita^ (with diabetes) mice 24. Thus, we hypothesized that the adverse effect of telomeres shortening on mitochondrial function might worsen inflammation, increase TNF-a levels, and accelerate kidney dysfunction. Our study indicates that TNF-a might mediate the negative relationship between LTL and kidney dysfunction from a clinical perspective. Further studies are required to clarify the details of these mechanisms, for example, epigenetic analysis. Epigenetics has previously been used to highlight a correlation between telomere length and decreased kidney function 39. Kidney function is reduced in CKD patients with shorter telomere length 4.

Regarding the last question, we found a more prominent association between shorter LTL and the risk of kidney dysfunction in participants with hypertension than that in participants without hypertension. Hypertension increases the risk of decreased eGFR, blood pressure is an important predictor of early renal function decline 40, 41, and oxidative stress and inflammation might play important roles in these outcomes 42. Although no studies have reported such results, a previous study showed that the relationship between LTL and CKD progression is stronger in smokers and those with diabetes who are more likely to have higher inflammatory and oxidative stress statuses 30. Thus, together with the previous study, our results suggest that renal function may not respond to LTL per se, but to the risk factors inflammation and oxidative stress, which are influenced by LTL shortening. LTL could be a useful predictor of renal dysfunction in patients with hypertension. In the future, Mendelian Randomisation may be helpful to explain the greater relationship between LTL and kidney dysfunction in hypertensive patients.

Our study has several strengths. First, our analysis focused on the relationship between LTL and kidney dysfunction in participants with hypertension, for which there are few related studies. Second, we analyzed the role of inflammatory stress in the LTL-CKD link and found that TNF-a mediates the effect between LTL and kidney dysfunction. However, our study has some drawbacks. This study included a small sample size and could not draw causal conclusions because of the study design. Future studies will expand the sample size and extend the follow-up period.

## Conclusion

LTL was negatively associated with the kidney dysfunction especially rapid decline in renal function during follow-up. TNF-a partially mediates the association between LTL and rapid decline in renal function. The association between LTL and kidney dysfunction is more pronounced in patients with hypertension. Therefore, LTL may be an important clinical predictor of kidney dysfunction in patients with hypertension. Future studies should be conducted using a larger population to better understand the role of LTL in changes in kidney function.

## Supplementary Material

Supplementary figures and table.

## Figures and Tables

**Table 1:** Baseline characteristics of participants by LTL quartiles.

	Total(n=262)	Q1 (n=65)	Q2 (n=66)	Q3 (n=65)	Q4 (n=66)	P
**Age, years**	54.3±9.7	54.1±10.1	54.2±10.3	54.5±9.5	54.4±9.1	0.998
**Male, n(%)**	78(29.8)	21(32.3)	19 (28.8)	21 (32.3)	17 (25.8)	0.816
**BMI, kg/m^2^**	26.2±4.1	26.1±4.4	26.0±4.3	26.3±4.0	26.5±4.0	0.878
**WHR**	0.95±0.14	0.95±0.02	0.95±0.02	0.94±0.02	0.98±0.27	0.454
**SBP, mmHg**	128.3±19.6	129.8±18.9	126.7±15.0	129.0±21.0	127.9±22.9	0.822
**DBP, mmHg**	76.2±10.2	78.0±9.2	75.3±11.0	76.8±9.5	74.9±11.0	0.294
**FPG, mmol/L**	6.6±2.0	6.6±2.4	6.4±1.4	7.0±1.9	6.5±2.2	0.397
**HbA1c, %**	5.9±1.1	6.0±1.3	5.8±0.8	6.1±1.2	5.7±0.9	0.134
**TC, mmol/L**	5.5±1.1	5.5±1.0	5.5±1.2	5.6±0.9	5.5±1.1	0.958
**TG, mmol/L**	1.4(1.0,2.1)	1.4 (1.1, 2.1)	1.3 (0.9, 2.4)	1.5 (1.1, 1.9)	1.5 (1.0, 2.2)	0.807
**HDL-C, mmol/L**	1.3±0.4	1.4±0.7	1.3±0.3	1.3±0.2	1.3±0.3	0.337
**LDL-C, mmol/L**	2.9±0.7	2.9±0.7	2.8±0.9	2.9±0.7	2.9±0.7	0.948
**eGFR, mL/min/1.73 m^2^**	96.9±14.3	93.3±15.8	101.0±13.6	97.5±12.2	95.7±14.4	0.015*
**ACR, mg/g Cr**	11.7(6.9,23.5)	8.9(5.4,16.7)	13.6 (6.9, 30.1)	12.3 (6.9, 25.9)	11.9 (7.6, 18.4)	0.139
**Microalbuminuria, n (%)**	48(18.3)	10 (15.4)	17 (25.8)	13 (20.0)	8 (12.1)	0.201
**Diabetes, n (%)**	158(60.3)	33 (50.8)	40 (60.6)	46 (70.8)	39 (59.1)	0.173
**Hypertension, n (%)**	81(30.9)	22 (33.8)	19 (28.8)	21 (32.3)	19 (28.8)	0.896
**Hyperlipidemia, n (%)**	164(62.6)	38 (58.5)	40 (60.6)	42 (64.6)	44 (66.7)	0.730
**Hyperuricemia, n (%)**	26(9.9)	8 (12.3)	9 (13.6)	5 (7.7)	4 (6.1)	0.416
**Obesity, n (%)**	77(29.4)	21 (32.3)	20 (30.3)	15 (23.1)	21 (31.8)	0.651
**TNF-a, pmol/L**	24.1±10.8	28.3±9.0	26.3±11.8	22.5±10.4	19.2±9.5	<0.001*
**IL-6, pg/mL**	3.2(2.0,4.6)	3.2 (1.9,4.5)	3.4(2.6,4.4)	2.8(1.8,4.1)	3.5(2.0,6.1)	0.096
**8OHdG, pg/mL**	38.6(20.7,58.3)	37.3(22.6,56.3)	38.6(16.6,65.1)	35.1(20.7,53.4)	43.8(26.6,64.4)	0.473
**SOD, U/mL**	60.6±18.6	64.5±16.5	65.0±19.4	58.6±16.0	54.4±20.6	0.002*
**GR, U/L**	6.4(4.8,9.6)	6.4(4.8, 9.6)	6.4(4.8, 9.6)	6.4(3.2,9.6)	6.4(3.2, 9.6)	0.813
**Lg(LTL), Z score**	0.00±1.00	-1.23±0.54	-0.32±0.15	0.29±0.20	1.26±0.59	<0.001*

Data are presented as mean±standard deviation or median (Q1, Q3) for skewed variables or proportion of participants (%), as appropriate. *Statistically significant LTL, leukocyte telomere length; Q, quartile; BMI, body mass index; WHR, waist-to-hip ratio; SBP, systolic blood pressure; DBP, diastolic blood pressure; FPG, fasting plasma glucose; HbA1c, glycated hemoglobin; TC, total cholesterol; TG, triglycerides; HDL-C, high-density lipoprotein cholesterol; LDL-C, low-density lipoprotein cholesterol; eGFR, estimated glomerular filtration rate; ACR, urine albumin-creatinine ratio; TNF-a, tumor necrosis factor-a; IL-6, interleukine-6; 8-OHdG, 8-hydroxy-2 deoxyguanosine; SOD, superoxide dismutase; GR, glutathione reductase

**Table 2:** The association between baseline LTL and the risk of albuminuria progression and/or rapid decline in renal function.

	Albuminuria progression(n=262)					Rapid decline in renal function(n=262)				Composite endpoint(n=262)					
	OR (95% CI)				P for trend	OR (95% CI)				P for trend	OR (95% CI)				P for trend
Model	Per 1SD	Q1 vs. Q4	Q2 vs. Q4	Q3 vs. Q4		Q3 vs. Q4	Q1 vs. Q4	Q2 vs.Q4	Q3 vs. Q4		Per 1SD	Q1 vs. Q4	Q2 vs.Q4	Q3 vs. Q4	
1	1.21 (0.85,1.74)	2.17 (0.82,6.20)	1.14 (0.38,3.46)	1.44 (0.50,4.31)	0.173	1.76 (0.67,4.84)	**10.76 (1.92,201.84)**	**8.87 (1.55,167.27)**	3.32 (0.41,68.34)	**0.004**	**1.42 (1.04,1.97)**	**3.12 (1.28,8.24)**	2.10 (0.83,5.64)	1.76 (0.67,4.84)	**0.013**
2	1.23 (0.85,1.78)	2.42 (0.90,7.03)	1.21 (0.40,3.71)	1.57 (0.54,4.78)	0.128	1.89 (0.71,5.27)	**11.33 (2.00,213.55)**	**9.00 (1.56,170.44)**	3.32 (0.40,68.53)	**0.004**	**1.45 (1.05,2.02)**	**3.46 (1.40,9.26)**	2.24 (0.88,6.06)	1.89 (0.71,5.27)	**0.008**
3	1.19 (0.81,1.74)	2.21 (0.79,6.65)	1.09 (0.34,3.46)	1.61 (0.54,4.99)	0.211	1.80 (0.66,5.08)	**7.72 (1.28,** **148.75)**	**6.73 (1.10,130.39)**	3.20 (0.38,67.13)	**0.029**	1.34 (0.96,1.89)	**2.86 (1.12,7.83)**	1.89 (0.72,5.26)	1.80 (0.66,5.08)	**0.036**
4	1.20 (0.82,1.79)	2.31 (0.81,7.03)	0.93 (0.29,3.03)	1.51 (0.50,4.73)	0.204	1.71 (0.62,4.87)	**7.57 (1.25,** **145.88)**	5.93 (0.92,117.02)	3.02 (0.35,63.64)	**0.031**	1.37 (0.97,1.96)	**2.96 (1.15,8.20)**	1.61 (0.60,4.56)	1.71 (0.62,4.87)	**0.036**

Model 1: not adjusted. Model 2: adjusted for age, sex. Model 3: adjusted for Model 2 +BMI,HbA1c,SBP,DBP,LDL-C,TG,UA. Model 4: adjusted for Model 3+ baseline ACR,baseline eGFR. LTL, leukocyte telomere length; Q, quartile; OR, odds ratio; CI confidence interval. Bold font indicates statistical differences.

**Table 3:** Mediation models of the association among TNF-a, LTL, and kidney outcome.

	Rapid decline in renal function		Composite endpoint(n=59)	
	Estimate (95%CI)	p value	Estimate (95%CI)	p value
All participants				
Total effect	0.059 (0.019,0.110)	**<0.001**	0.058 (0.008,0.110)	**<0.001**
Indirect effect	0.013 (0.003,0.020)	**0.040**	0.009 (-0.003,0.020)	0.200
Direct effect	0.046 (0.006,0.090)	**<0.001**	0.048 (-0.004,0.100)	0.080
Prop. Mediated	0.224 (0.040,0.680)	**0.040**	0.181 (-0.045,1.570)	0.200
Participants with hypertension				
Total effect	-0.002 (-0.164,0.200)	0.960	0.147 (0.047,0.230)	**<0.001**
Indirect effect	-0.001 (-0.080,0.050)	0.800	0.005 (-0.023,0.030)	0.640
Direct effect	-0.001 (-0.116,0.160)	0.920	0.141 (0.050,0.230)	**<0.001**
Prop. Mediated	0.206 (0.000,0.540)	0.040	0.030 (-0.135,0.330)	0.640

Models were adjusted for age, sex,BMI,HbA1c,SBP,DBP,LDL-C,TG,UA,baseline ACR and baseline eGFR. Bold font indicates statistical differences.

**Table 4:** The association between per 1 SD decrease of baseline LTL and the risk of albuminuria progression and/or rapid decline in renal function in participants with or without hypertension

	Albuminuria progression(n=262)			Rapid decline in renal function(n=262)			Composite endpoint(n=262)		
	OR (95% CI), P		P for interaction	OR (95% CI), P		P for interaction	OR (95% CI), P		P for interaction
Model	With hypertension	Without hypertension		With hypertension	Without hypertension		With hypertension	Without hypertension	
1	1.85(0.97,3.89) 0.075	0.99(0.65,1.54) 0.995	0.131	**5.49(1.91,23.39) 0.006**	1.44(0.82,2.61) 0.206	0.051	**2.77(1.45,6.04)** **0.004**	1.11(0.76,1.62) 0.580	**0.024**
2	1.90(0.96,4.12) 0.076	1.02(0.66,1.60) 0.908	0.164	**7.50(2.30,40.46) 0.004**	1.44(0.82,2.60) 0.209	**0.037**	**2.99(1.52,6.73)** **0.003**	1.13(0.78,1.66) 0.516	**0.026**
3	2.06(0.98,4.77) 0.066	1.01(0.65,1.60) 0.937	0.172	**11.27(2.33,182.38)** **0.017**	1.33(0.73,2.45) 0.349	**0.047**	**2.87(1.42,6.55) 0.005**	1.06(0.72,1.57) 0.754	**0.034**
4	2.00(0.92,4.76) 0.090	1.02(0.65,1.65) 0.905	0.185	**49.07(3.72,211.12)** **0.041**	1.32(0.69,2.58) 0.391	**0.045**	**3.10(1.48,7.52)** **0.005**	1.08(0.72,1.63) 0.691	**0.036**

Model 1: not adjusted. Model 2: adjusted for age, sex. Model 3: adjusted for Model 2 +BMI,HbA1c,SBP,DBP,LDL-C,TG,UA. Model 4: adjusted for Model 3+ baseline ACR,baseline eGFR. LTL, leukocyte telomere length; OR, odds ratio; CI confidence interval. Bold font indicates statistical differences.
