# Supplementary material for: Tumor necrosis factor-alpha mediates the negative association between telomere length and kidney dysfunction
Source: Int J Med Sci. 2023 Sep 25;20(12):1592–9. doi: 10.7150/ijms.87254 (PMC10583187; doi:10.7150/ijms.87254)
Supplement: Supplementary file 1 — Supplementary figures and table. [file ijmsv20p1592s1.pdf]

Supplementary Table 1 Mediation models of the association among inflammation and oxidative stress indicators, LTL, and kidney outcome

	Rapid decline in renal function		Composite endpoint	
	Estimate(95%CI)	p value	Estimate(95%CI)	p value
SOD				
Total effect	0.057(0.017, 0.100)	**<0.001**	0.058(0.008,0.110)	**<0.001**
Indirect effect	0.005(-0.002,0.020)	0.200	0.008(-0.001,0.020)	0.200
Direct effect	0.052(0.010,0.100)	**<0.001**	0.050(-0.000,0.100)	0.120
Prop. Mediated	0.119(-0.034,0.420)	0.200	0.148(-0.039,1.090)	0.200
GR				
Total effect	0.056(0.014,0.100)	**<0.001**	0.0590(0.005,0.110)	**<0.001**
Indirect effect	0.000(-0.003,0.000)	0.880	-0.000(-0.003, 0.000)	0.760
Direct effect	0.056(0.016,0.100)	**<0.001**	0.0596(0.010,0.110)	**<0.001**
Prop. Mediated	0.001(-0.132,0.080)	0.880	-0.003(-0.828,0.040)	0.760
8OHdG				
Total effect	0.056(0.014,0.100)	**<0.001**	0.057(0.009,0.110)	**0.040**
Indirect effect	0.000(-0.005,0.010)	0.920	0.000(-0.009,0.010)	0.960
Direct effect	0.056(0.017,0.100)	**<0.001**	0.057(0.008,0.110)	**<0.001**
Prop. Mediated	0.002(-0.293,0.100)	0.920	0.009(-0.617,0.710)	0.920
IL-6				
Total effect	0.049(0.006,0.080)	**0.040**	0.057(0.007,0.110)	**0.040**
Indirect effect	-0.005(-0.014,0.000)	0.080	-0.006(-0.021,0.000)	0.120
Direct effect	0.054(0.008,0.090)	**<0.001**	0.064(0.015,0.120)	**<0.001**
Prop. Mediated	-0.096(-0.557,0.040)	0.120	-0.094(-1.221,0.040)	0.160

Models were adjusted for age, sex,BMI,HbA1c,SBP,DBP,LDL-C,TG,UA,baseline ACR and baseline eGFR.

Bold font indicates statistical differences.

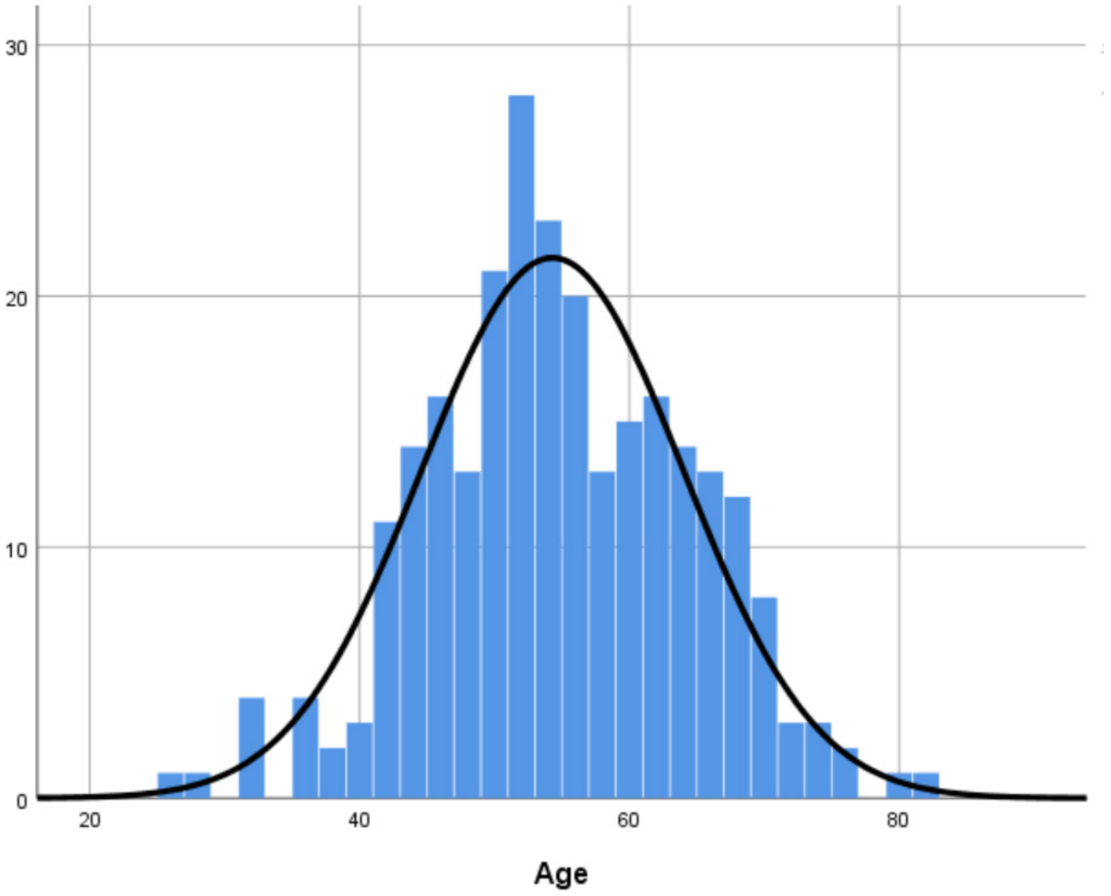

Supplementary Figure 1 Histogram of age in the study

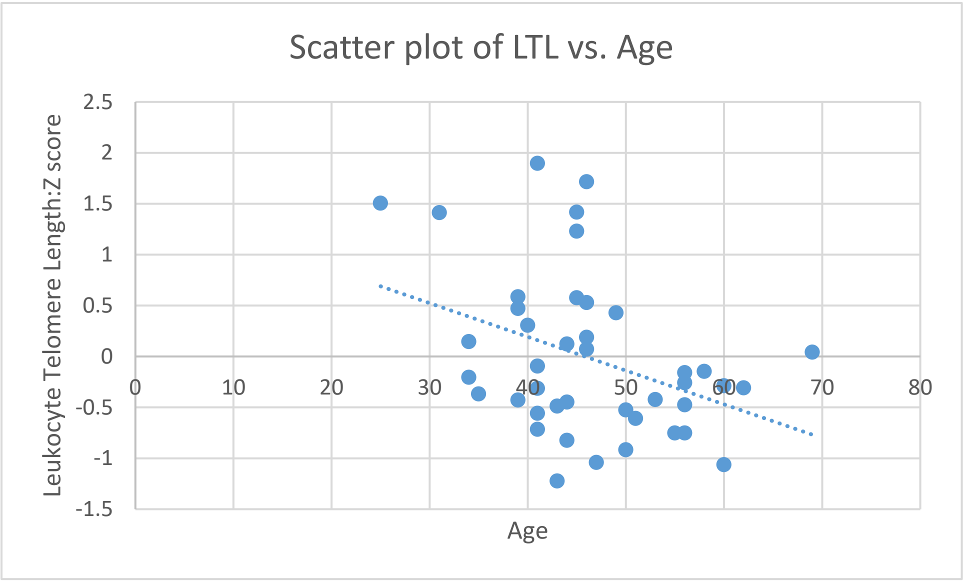

Supplementary Figure 2 Scatter plot of LTL *vs.*Age in 42 healthy participants

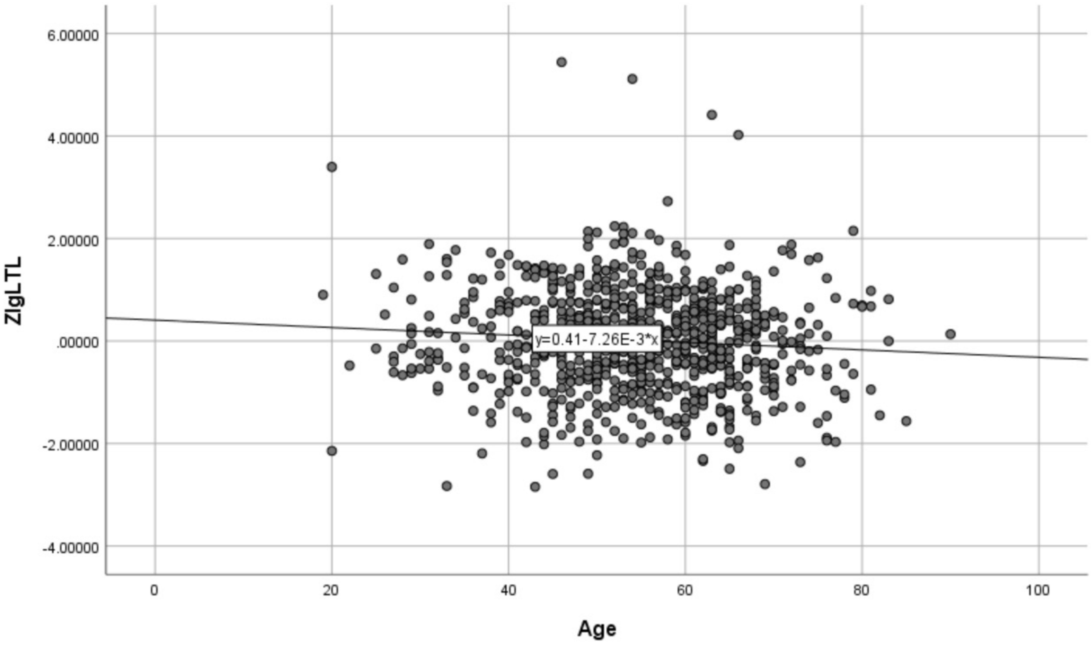

Supplementary Figure 3 Scatter plot of LTL *vs.*Age with a total sample size of 832
